# Neuropathogenesis of Encephalitic Alphaviruses in Non-Human Primate and Mouse Models of Infection

**DOI:** 10.3390/pathogens14020193

**Published:** 2025-02-14

**Authors:** Caitlin M. Woodson, Shannon K. Carney, Kylene Kehn-Hall

**Affiliations:** 1 Department of Biomedical Sciences and Pathobiology, Virginia-Maryland College of Veterinary Medicine, Virginia Polytechnic Institute and State University, Blacksburg, VA 24061, USA; woodsonc@vt.edu (C.M.W.); carney1@vt.edu (S.K.C.); 2 Center for Emerging, Zoonotic, and Arthropod-Borne Pathogens, Virginia Polytechnic Institute and State University, Blacksburg, VA 24061, USA

**Keywords:** Venezuelan equine encephalitis virus, eastern equine encephalitis virus, western equine encephalitis virus, sequelae, animal models

## Abstract

Encephalitic alphaviruses, including eastern, Venezuelan, and western equine encephalitis virus (EEEV, VEEV, and WEEV, respectively) are New World alphaviruses primarily transmitted by mosquitos that cause debilitating and lethal central nervous system (CNS) disease in both humans and horses. Despite over one hundred years of research on these viruses, the underpinnings of the molecular mechanisms driving virally induced damage to the CNS remain unresolved. Moreover, virally induced encephalitis following exposure to these viruses causes catastrophic damage to the CNS, and survivors of infection often suffer from permanent neurological sequelae as a result of sustained neuroinflammation and neurological insults encountered. Animal models are undoubtedly invaluable tools in biomedical research, where physiologically relevant models are required to study pathogenesis and host–pathogen interactions. Here, we review the literature to examine nonhuman primate (NHP) and mouse models of infection for EEEV, VEEV, and WEEV. We provide a brief overview of relevant background information for each virus, including geography, epidemiology, and clinical disease. The primary focus of this review is to describe neuropathological features associated with CNS disease in NHP and mouse models of infection and compare CNS invasion and neuropathogenesis for aerosol, intranasal, and subcutaneous routes of exposure to EEEV, VEEV, and WEEV.

## 1. Introduction

**Figure 1:**
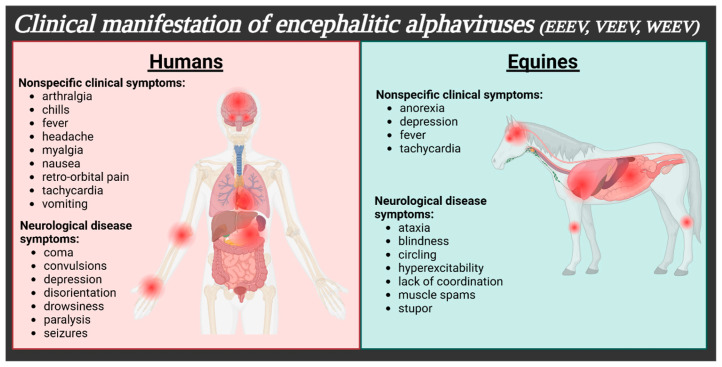
Overview of clinical disease of encephalitic alphaviruses in humans and equines. Humans infected with encephalitic alphaviruses may present with nonspecific clinical symptoms including arthralgia, chills, fever, headache, myalgia, nausea, retro-orbital pain, and vomiting. Acute symptoms may resolve following activation of the host immune system or may progress to encephalitic disease. Neurological disease symptoms in humans include coma, convulsion, depression, disorientation, drowsiness, paralysis, and/or seizures. Infection with encephalitic alphaviruses in equines may present with nonspecific clinical symptoms including loss of appetite/anorexia, depression, fever, and tachycardia. Encephalitic disease symptoms include ataxia, blindness, circling, hyperexcitability, lack of coordination, muscle spasms, and/or stupor. Created in BioRender. Woodson, C. (2025) https://BioRender.com/o80a348 (accessed on 17 January 2025).

Encephalitic alphaviruses, including eastern equine encephalitis virus (EEEV), Venezuelan equine encephalitis virus (VEEV), and western equine encephalitis virus (WEEV), are arboviruses that pose a significant threat to both human and equine health. As members of the genus *Alphavirus* within the *Togaviridae* family, these positive-sense single-stranded RNA viruses are primarily found in the Americas (North, Central, and/or South) and are transmitted by mosquitoes from the *Aedes*, *Culex*, *Psorophora*, and *Culiseta* genera, among other vectors [1]. Infection with encephalitic alphaviruses can be asymptomatic or cause mild, febrile, flu-like illness, which is usually self-limiting. However, in severe cases, infection can rapidly progress to invasion of the brain and spinal cord, resulting in significant neurological disease, morbidity, and mortality (Figure 1). Survivors of neurological disease often suffer from chronic neurological sequelae including depression, disorientation, coma, convulsions, seizures, paralysis, intellectual disability, and/or behavioral changes, all of which can be permanent and/or debilitating (Table 1). From a public health perspective, the institutionalized care and the medical and financial resources required to care for survivors of viral encephalitis is an enormous burden to patients, caregivers, and the healthcare system in general. As a result of globalization, climate change, and other contributing factors, outbreaks and incidences of viral encephalitis continue to rise [2,3]. Currently there are no approved vaccines or therapeutics for EEEV, VEEV, or WEEV infection, exacerbating the challenge of preventing and managing outbreaks and highlighting the need for continued research and development of effective therapeutics and vaccines against encephalitic alphaviruses.

Since these viruses are easily aerosolized, have a low infectious dose, and induce high morbidity upon exposure, EEEV, VEEV, and WEEV must all be handled under biosafety level 3 conditions, requiring specialized facilities, equipment, and training [1,4,5]. Moreover, EEEV and VEEV are classified as select agents by the Department of Health and Human Services (HHS) due to concerns over dual-use and bioweapon potential and lack of medical countermeasures available [3]. As such, entities working with select agents must register and have oversight by the Centers for Disease Control and Prevention (CDC). VEEV is an overlap select agent, with consideration from the United States Department of Agriculture (USDA) as well [6]. Thus, working with these viruses in the lab is challenging and highly demanding on time and resources.

**Table 1:** Overview of EEEV-, VEEV-, and WEEV-specific clinical features of disease, mortality, and neurological sequelae observed in humans.

Virus	Clinical Symptoms	Neurological Symptoms	% Mortality	% Survivors w/Sequelae	Neurological Sequelae	Refs.
EEEV	fever, headache, malaise, myalgia, nausea, and/or vomiting	behavioral changes, coma, and/or drowsiness	30–75%	50–90%	behavioral changes, convulsions, intellectual disability, paralysis, and/or seizures	[1,4,7,8]
VEEV	arthralgia, chills, fever, headache, malaise, myalgia, photophobia, retro-orbital pain, vomiting, and/or diarrhea	ataxia, depression, disorientation, drowsiness, coma, confusion, convulsions, paralysis, and/or seizures	<1% (up to 10% of adult neurological cases, 35% children)	4–14%	altered personality, coma, confusion, convulsions, depression, emotional instability, photophobia, intellectual disability, paralysis, epilepsy, recurrent headaches, seizures, and/or somnolence	[1,4,7]
WEEV	fever, headache, malaise, myalgia, nausea, and/or vomiting	agitation, coma, confusion, drowsiness, light sensitivity, and/or seizures	3–15%	15–30%	coma, confusion, visual disturbances, emotional instability/behavioral changes, intellectual disability, photophobia, seizures, somnolence, taste distortion, spastic paresis, and/or respiratory distress	[1,4,7]

Animal models play a critical role in research, especially in evaluating potential antiviral and vaccine candidates. Under the Animal Rule, the FDA may grant approval for drugs or biological products based on well controlled animal studies when human challenge studies are not ethical or feasible [9,10]. Since there are limited clinical data of encephalitic alphavirus infection in humans available, well characterized animal models that recapitulate severe human disease are required to study the pathogenesis of encephalitic alphaviruses [10]. Encephalitic clinical symptoms in humans include abrupt onset of fever, intense headache, irritability, restlessness, drowsiness, anorexia, nausea, vomiting, diarrhea, cyanosis, convulsions, and/or coma [11]. Therefore, an appropriate animal model for encephalitic alphaviruses should present clinically as a biphasic course of disease with viral replication in the periphery prior to invasion into the central nervous system (CNS) with or without neurological symptoms of disease [7,10]. A better understanding of the early host cell targets of encephalitic alphaviruses may contribute to our understanding of viral pathogenesis and lead to the development of more effective vaccines and antiviral and/or host factor targeted therapeutics.

In this review, we aim to assess the neuropathogenesis currently described in the literature for EEEV, VEEV, and WEEV. This review is not exhaustive; rather, it is meant to provide a broad overview of each virus and identify similarities and differences in neuropathogenesis, neuropathology, and neurological sequelae, focusing on non-human primate (NHP) and mouse models of encephalitic alphaviruses. We will also discuss relevant host-factors that have been identified thus far to play a role in neuropathogenesis. NHP and mouse models were chosen in particular because NHPs are the closest model to humans and mice because they manifest severe neurological disease and are readily accessible by researchers. Moreover, this review will focus on the three most commonly utilized routes of exposure including subcutaneous, intranasal, and aerosol routes of exposure. Other exposure routes, including intravenous, intracranial, and intraperitoneal routes of infection, have also been described for encephalitic alphaviruses but will not be discussed in this review. The rationale for not including other routes of infection is because subcutaneous exposure mimics natural infection via mosquito bite and aerosol and intranasal exposure are considered the gold standard for testing therapeutics and vaccines against encephalitic alphaviruses. The development of well characterized and physiologically relevant animal models is critical for antiviral and vaccine testing, as well as for determining mechanisms that contribute to neurological disease and sequelae.

## 2. Overview of Neuroinvasion and Neurovirulence

**Figure 2:**
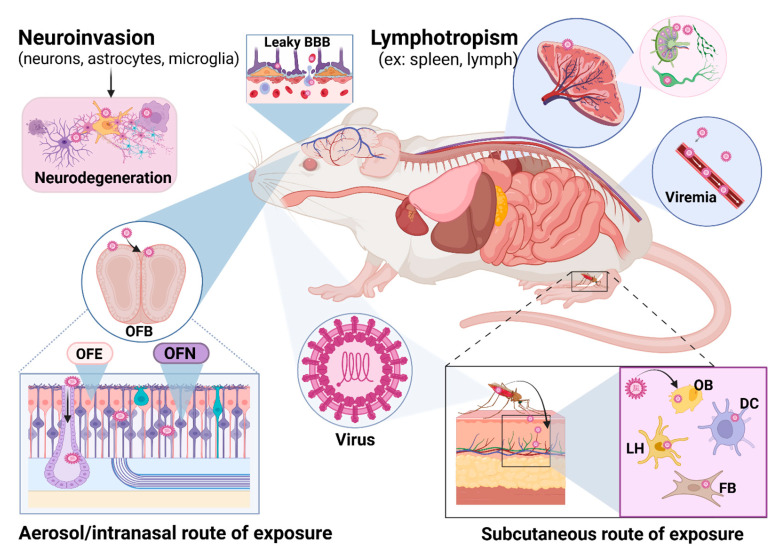
Overview of direct (aerosol/intranasal) or indirect (subcutaneous) routes of infection and virus dissemination in the laboratory mouse. Following aerosol or intranasal exposure of encephalitic alphaviruses, infection is established within the OFB, particularly the OFE and OFN. The subcutaneous route of exposure results in infection of DC, FB, LH, and/or OB cells which travel to lymphotropic regions via the bloodstream to establish infection in peripheral tissues, including the spleen and lymph nodes; however, EEEV is notably less lymphotropic as compared to VEEV and WEEV [23]. The virus invades the CNS through multiple and/or simultaneous routes, including entering through the CVOs of the brain, which naturally lack a BBB. However, the BBB is still intact upon CNS entry and does not lose integrity until peak viral replication in the brain. CNS invasion can also occur following establishment of viremia via hematogenous routes through multiple entry sites that are not exclusive to CVOs as well as through caveola-mediated transcytosis. OFB: olfactory bulb, OFE: olfactory epithelium, OFNs: olfactory neurons, BBB: blood–brain barrier, CVOs: circumventricular organs, DCs: dendritic cells, FBs: fibroblasts, OBs: osteoblasts, LHs: Langerhans cells. Created in BioRender. Woodson, C. (2025) https://BioRender.com/z06t406 (accessed on 17 January 2025).

Exposure to encephalitic alphaviruses has the potential to result in severe consequences including death or permanent neurological sequelae. In this section, an overview of the process of neuroinvasion and neurovirulence is provided; however, viral-specific mechanisms are described in more detail in the following sections. Viral strain and challenge dose, as well as route of exposure, are important factors to consider when studying encephalitic alphaviruses. A graphical overview of direct and indirect routes of exposure in a mouse model of infection is shown in Figure 2. Subcutaneous exposure mimics natural infection via a mosquito vector. Upon an infected vector taking a blood meal, infectious virions are transferred to the bloodstream and lymphoid tissues from permissive cell types including dendritic, fibroblast, osteoblast, and Langerhans cells to amplify viral replication and dissemination [12]. Viremia is usually, but not always, observed in this route of exposure. Viral entry is mediated by the very-low-density lipoprotein receptor (VLDLR) for EEEV [13,14,15], low-density lipoprotein receptor class A domain-containing protein 3 (LDLRAD3) for VEEV [16], and either protocadherin-10 (PCDH10) or VLDLR for WEEV [17,18]. Activation of the host immune response and infiltrating leukocytes and neutrophils, along with viral replication, results in disruption of the BBB, further adding insult to injury [19]. However, these viruses are capable of penetrating the CNS regardless of the BBB permeability [20], all but ensuring neuroinvasion once infection is established within the host. Direct exposure to encephalitic alphaviruses through either aerosol or intranasal routes of infection typically, but not always, results in heavy involvement of the olfactory bulb [7]. Virions penetrate the olfactory neuroepithelium and olfactory neurons to establish infection [21]. Once inside the CNS, the virus targets neurons, astrocytes, microglia, and oligodendrocytes, among other cell types. The combination of viral replication, activation of the host immune response, and release of proinflammatory cytokines and chemokines that result in altered cell signaling pathways all contribute to neurodegeneration and cell death within the infected host [19,22] (Figure 2).

**Figure 3:**
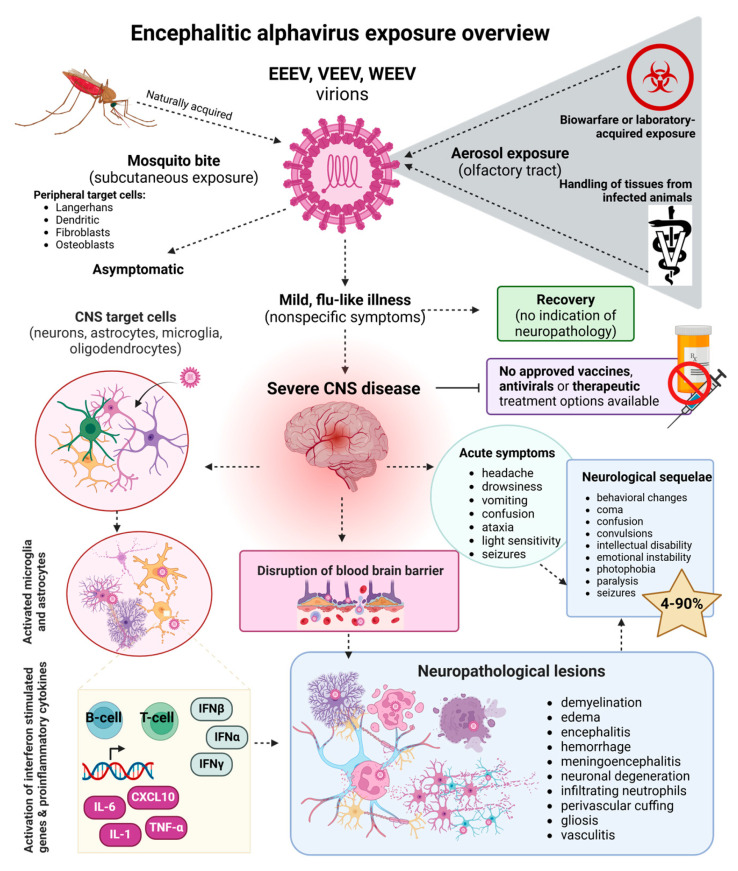
Summary of encephalitic alphavirus exposure and resulting neuropathology and neurological sequelae. Exposure to EEEV, VEEV, and WEEV can occur naturally through the bite of an infected vector (ex: mosquito) or through aerosol exposure as a result of handling infectious tissues or through laboratory or biowarfare exposure. While a majority of infections in humans are asymptomatic, disease can manifest as mild, flu-like illness that either self resolves or progresses to severe CNS disease. Importantly, there are no FDA-approved vaccines, antivirals, or therapeutic treatments available for infected patients. Severe disease presents with various acute symptoms including but not limited to headache, drowsiness, vomiting, confusions, ataxia, light sensitivity, and/or seizures. The virus targets specific CNS cell types, including neurons, astrocytes, microglia, and oligodendrocytes, among others. Infection of these cells prompts activation of microglia and astrocytes which stimulates transcription of interferon stimulated genes and numerous proinflammatory cytokines. During peak viral replication within the brain, the BBB becomes compromised and provides yet another route for CNS insults. The combinatorial effects of viral replication and sustained inflammation in response to infection results in neurodegeneration and severe neuropathological lesions including demyelination, edema, encephalitis, hemorrhage, meningoencephalitis, infiltrating neutrophils, perivascular cuffing, gliosis, and/or vasculitis, among others, all of which contribute to disease outcome. Neurological sequelae as a result of severe CNS disease include permanent mood and behavioral changes, coma, confusion, convulsions, intellectual disability, emotional instability, photophobia, paralysis, and/or seizures, in as high as 90% of survivors of infection, severely impacting the quality of life for surviving patients. Created in BioRender. Woodson, C. (2025) https://BioRender.com/w83r176 (accessed on 17 January 2025).

Following CNS invasion, regardless of exposure route, the host immune system must respond appropriately and promptly in order to clear the virus and offset consequences of inflammatory mediators released from activated astrocytes, neurons, and microglial cell populations [24]. Uncontrolled, sustained activation of proinflammatory markers results in neuronal loss and degeneration as activated microglia and astrocytes release proinflammatory cytokines and chemokines as well as reactive oxygen and nitrogen species [25], which ultimately contribute to clinical and pathological outcomes (Figure 3). Microglia cells are the sole brain resident immune cell and enhance neurogenesis as well as synaptic remodeling [26]. Importantly, microglia involvement is critical for surviving encephalitis [27]. Neurons and glial cells release type I interferon, which directly modulates viral replication in the CNS. Astrocytes play a major role in maintaining the tight junctions of the BBB, as well as regulating neuroinflammation and neuroplasticity [28]. Neutrophils play an essential role in killing invading pathogens, and the presence of infiltrating neutrophils following infection with encephalitic alphaviruses is associated with an increase in the severity of neuropathology [29]. Following detection of infected neurons, the host innate immune response triggers transcription of antiviral response genes including interferon beta (*IFNβ*) and interferon gamma (*IFNγ*); inflammatory markers such as cytokines C-X-C motif chemokine 10 (*CXCL10*), tumor necrosis factor alpha (*TNFα*), interleukin 6 (*IL-6*), and interleukin 1 (*IL-1*); and genes encoding the adhesion proteins, intercellular adhesion molecule-1 (ICAM-1), and vascular cell adhesion molecule 1 (VCAM-1), which facilitate attachment of leukocytes to endothelial cells lining the BBB [19,22]. Stimulation of adaptive immunity, including B- and T-cells, occurs following interaction with antigen-presenting cells and cytokine signaling [12]. Immune cells migrate from lymphoid tissues to sites of infection in order to contribute to local inflammation, dampen viral replication, and contribute to the clearance of infected cells [12]. Upon viral invasion of the CNS and disruption of the BBB, inflammatory mediators are stimulated, ultimately contributing to bystander damage and enhanced neuropathology. Commonly described neuropathological features, aside from the obvious encephalitis, include loss of neurons through demyelination, edema, hemorrhage, infiltrating neutrophils, perivascular cuffing, gliosis, and vasculitis [30]. These neurological insults may have permanent damage that presents as confusion, coma, emotional instability, intellectual disability, photophobia, paralysis, and seizures, among others, highlighting the urgent need for the development of efficacious therapeutics and vaccines to prevent or subdue catastrophic damage within the CNS.

## 3. Eastern Equine Encephalitis Virus (EEEV)

### 3.1. Geography, Epidemiology, Clinical Disease

EEEV is found primarily in eastern North America on the Gulf and Atlantic coasts, in some midwestern states around the Great Lakes, and in southeastern Canada [31]. Enzootic transmission of EEEV is maintained between passerine birds and mosquitoes particularly in and around freshwater hardwood swamps [32]. Importantly, humans and horses serve as dead-end hosts and do not develop significant viremia, whereas birds serve as amplification hosts in the transmission cycle [32]. EEEV is the most lethal of the encephalitic alphaviruses, with a case fatality rate of 30–75% in humans and with 50–90% of all survivors suffering from neurological sequelae [1,5,8]. Case fatality for equines is significantly higher, nearing 100 percent [33]. EEEV is also one of the most acutely virulent arboviruses in the Americas.

Limited outbreaks of EEEV occur, with an average of 11 human cases reported annually (CDC). In the US, there have been a total of 121 human EEEV cases reported between 2003 and 2016, with 119 of these cases being classified as neuroinvasive and 110 caused encephalitis or meningoencephalitis, resulting in 50 deaths [34]. In 2019, there was an outbreak of EEEV in humans and horses in the northeast US, where 38 human and 26 horse cases were reported, along with 19 deaths and neurological sequelae observed in survivors [2,35]. Cases were primarily reported in counties which had previously reported EEEV-positive humans, horses, or mosquitoes before 2019. Moreover, there were 19 human cases of EEEV, all neuroinvasive, in nine states along the northeast reported by the CDC in 2024, with at least two cases resulting in fatality of an elderly man and a 41-year-old man with no underlying conditions [36,37]. Humans with comorbidities including obesity and preexisting conditions such as autoimmune disease, cancer, Hepatitis C, and Parkinson’s disease (PD) may have more severe clinical outcomes following infection with EEEV, including death [35]. MRIs obtained from four admitted patients during the 2019 EEEV outbreaks revealed hyperintensity within the cerebrum, cerebellum, brainstem, basal ganglia, and thalamus, and three patients succumbed to severe disease [35].

Infection with EEEV produces a biphasic illness that is characterized by an early self-limiting replication phase in peripheral tissues followed by a commonly fatal CNS phase [38]. After an incubation period of approximately 3 to 10 days, clinical symptoms present nonspecifically with fever, malaise, headache, muscle aches, nausea, and vomiting [39]. Clinical symptoms observed during the 2019 outbreak included fever, headache, confusion, stupor, dysarthria, coma, shock, seizures, and paralysis [35]. Juveniles appear to be more prone to developing encephalitis than adults, likely due to age-dependent factors in adult neurons that can help suppress viral replication once inside the CNS [40]. Mature neurons express apoptosis inhibitors, which can help make them more resistant to virus-induced apoptosis and neuronal death [24]. In a study where 41% of EEEV-infected patients suffered from seizures, it was found that 100% of children had seizures compared with 29% of adults [41]

During peripheral infection, as in the case of a mosquito bite, EEEV targets mesenchymal lineage cells, including osteoblasts, fibroblasts, and myocytes, to establish viral amplification in the periphery while avoiding triggering the host innate immune response [23]. This is in contrast to VEEV, which replicates to high levels in lymphoid tissues. Metaphyseal osteoblasts as an early site of replication may explain the high-titer viremia and high incidences of neuroinvasion observed in young animals, as that particular cell type is found near the end of long bones and is active in growing animals [38], which may contribute to infants being more susceptible to developing severe disease.

Upon CNS invasion in humans, EEEV demonstrates a tropism for neurons and has been reported to induce neuronal loss, neuronophagia, perivascular cuffs, focal and diffuse accumulations of inflammatory cells, and leptomeningitis [42]. EEEV displays early involvement of the basal ganglia and thalami, with focal lesions observed on both CT and MRI scans [43]. Autopsy of brains reveals edema, leptomeningeal vascular congestion, hemorrhage, and encephalomalacia with widely distributed small necrotic foci. Histopathologic findings suggest complex and progressive patterns of brain injury, with vascular, neuronal, and glial components observed, including arteriolar and venular inflammation, small-vessel thrombosis, perivascular cuffing, meningeal infiltrate, neuronal destruction, neuronophagia, focal necrosis, spotty demyelination, and glial nodules [44]. Cortical atrophy was also significantly present in the brains of patients who died later in the course of disease, a long-term consequence of severe neuronal damage and loss that can lead to cognitive and motor impairments after acute infection has passed [44,45]. In horses examined postmortem following euthanasia, EEEV was isolated from the CNS, with neurons, astrocytes, oligodendrocytes, microglia, and neutrophils showing infection with EEEV via antigen staining. Disease was characterized by lesions in the cerebral cortex, thalamus, hypothalamus, and mesencephalon that displayed perivascular cuffing, marked gliosis, neuronophagia, and multifocal micro abscesses [46].

### 3.2. Animal Models, CNS Disease, and Neuropathology

Animal models are important tools for studying the neuropathogenesis of EEEV and for evaluating potential antivirals and vaccine candidates. Several NHP and mouse models for EEEV have been characterized (Table 2), however not to the extent of which VEEV or WEEV have been studied. Mice and guinea pigs have often been used for studying basic viral replication and immune responses following EEEV infection, while NHP models provide a view of how the virus may be behaving in humans [7].

Several recent studies have examined viral dissemination and pathogenesis in NHPs infected with EEEV. Comprehensive pathological examination of a macaque model of aerosolized EEEV revealed that viral tropism was restricted primarily to neurons and viral particles were present within axons of neurons and throughout extracellular spaces. Interestingly, active viral replication seemed to be limited to regions of the brain that were proximal to the olfactory tract, suggesting that after aerosol challenge, EEEV may replicate in the olfactory bulb and be transported to distal regions of the brain using neuronal axonal transport to facilitate cell-to-cell spread [47]. In this model, mild to moderate necrotic and inflammatory lesions were observed, with necrotic lesions characterized by neuronal degeneration, satellitosis, necrosis, and vacuolation of the neuropil. Viral RNA and proteins were observed in the amygdala, hippocampus, thalamus, and hypothalamus. However, interestingly, minimal to no microscopic lesions were observed in the midbrain, forebrain, and hindbrain. Additionally, viral RNA was detected in the cervical spine but was absent of pathological lesions aside from minimal inflammation. These results suggest that aerosol exposure of EEEV results in rapid and direct spread of virus from the olfactory bulb into the brain. The minimal pathology but abundant infection of neurons suggests that neuropathogenesis may be a result of neuronal dysfunction as opposed to neuron cell death [47]. Another aerosol study in macaques implanted with telemetry monitoring devices showed that disruption in electroencephalography (EEG) and intracranial pressure (ICP) were indicators of lethal infection [49], suggesting that EEG and ICP are valuable and quantifiable data points that could provide insight into correlates of clinical disease. The rise in ICP in encephalitis may be broadly attributed to the diffuse swelling of parenchymal tissue. Necropsy of brains from lethal infections revealed exponential increases in expression of traumatic brain injury genes [leukemia inhibitory factor (*LIF*), matrix metalloproteinase-9 (*MMP-9*)] as well as inflammatory cytokines and chemokines [IFNγ, CXCL10, monocyte chemoattractant protein-1 (MCP-1), interleukin 8 (IL-8), IL-6] [49], which implies that altered host cell signaling contributes to fatal outcomes.

Common marmosets have also been utilized to study EEEV pathogenesis via the intranasal route of infection. This model resulted in lethal encephalitis, leukocytosis attributed to neutrophilia and lymphocytosis, neuronal loss, neuronophagia, and leptomeningitis [42], which have all been described in human cases of EEEV. Upon infection with neurotropic viruses, neutrophils are deployed from the bone marrow to the CNS where they interact with chemokine ligands, increase permeability of the BBB, and drive neuropathology through demyelination [29]. As such, the common marmoset appears to be a clinically relevant model for studying EEEV pathogenesis in NHPs, especially in terms of damage encountered by infiltrating neutrophils.

Differences in tissue tropism have been described for NHPs infected with EEEV subcutaneously as opposed to aerosol. In the subcutaneous infection model, much higher levels of viremia were observed, while in the aerosol model, despite a high infectious dose, viremia was either transient or undetectable; however, pathology was observed in the brains of three out of four NHPs post-infection [4]. Histologic lesions were observed in the brains of the three animals that succumbed to infection, along with acute inflammation in the cerebrum, cerebellum, and brain stem neuropil; chronic-active inflammation in the meninges, choroid plexus, and cerebral neuropil; gliosis in the brain stem; chronic-active perivascular cuffing in the cerebrum, cerebellum, and the brain stem; and hemorrhage in the cerebrum and brain stem neuropil [4]. Chronic-active inflammation in these regions suggests that infection persists beyond the acute phase, leading to long-term immune responses that may contribute to manifestation of long-term neurological sequelae. Specifically, this indicates that the virus has caused inflammatory damage to both the protective layers and fluid systems of the brain [4]. Perivascular cuffing is a hallmark of inflammation where immune cells surround blood vessels in the brain, suggesting that the virus induced the vasculitis and hemorrhage observed in the CNS of these animals [4]. Moreover, associations between perivascular cuffing and neuroinflammatory diseases such as multiple sclerosis (MS) and lupus have been described, with increased prominence of perivascular cuffs being correlated with decreased cognitive abilities in these patients [54].

Multiple mouse strains have been used to study EEEV, including both inbred and outbred strains of mice. A comprehensive comparison of exposure routes and the corresponding clinical disease and neuropathology induced have been well described previously [50,51]. These studies indicated that different inoculation methods of EEEV in mice result in different clinical courses of disease and corresponding neuropathology.

Aerosol exposure of mice to EEEV results in severe meningoencephalitis, including neuronal cell death and degeneration, with the presentation of overt clinical signs of disease coinciding with peak viral load in tissues examined [53]. Infectious virus can be detected in the brain within 6 h of inoculation via aerosol, likely due to the virus having more direct contact with olfactory neurons, leading to rapid invasion of the olfactory bulb. The presence of pyknotic and karyorrhectic debris and swelling in the hippocampus suggests that EEEV directly infects and destroys neurons through apoptotic means, which could impair critical brain functions like memory and learning [55]. Enlarged perivascular spaces and signs of cell degeneration and apoptosis were observed in the brain, particularly in the piriform cortex and hippocampus, which points to vascular inflammation and peripheral immune responses, suggesting that the virus may contribute to the disruption of BBB integrity [56,57]. Lesions were mostly bilateral, though some animals showed more severe effects on one hemisphere. The cortex and caudate putamen were primarily affected, with occasional involvement of more caudal brain regions. Lesions in the olfactory bulb correlated with brain hemisphere damage, and signs of apoptosis were observed in the olfactory epithelium, which suggests that the virus enters the brain via the olfactory route for the aerosol route of inoculation, despite also establishing infection in respiratory tissues [53].

In intranasally infected mice, EEEV was detected in both the blood and brain homogenates by day 1 post-infection (d1 pi), with viremia peaking and brain titers rising by d2 pi [50], suggesting a potential vascular route for neuroinvasion. Pathological assessments of intranasally infected mice on days 2 and 3 pi identified virus present in the olfactory epithelium but not the respiratory epithelium, olfactory bulb, or neurons in the piriform cortex of the cerebrum [51]. Cell death in virus-positive tissue was characterized by pyknosis and eosinophilic cellular and karyorrhectic debris without inflammation and presumably suggested apoptosis as the mechanism [51]. As the disease progressed, the viral antigen was noted multifocally in the nasal cavity and teeth as well as diffusely throughout the olfactory bulb, frontal cortex, midbrain, cerebellum, brain stem, and spinal cord [51]. Mild leptomeningitis with occasional perivascular cuffing was also noted [51]. Interestingly, the aerosol route of exposure resulted in earlier neuroinvasion as compared to the intranasal route of exposure in EEEV-infected mice [51]. The viral antigen was detected in the olfactory nerve and olfactory bulb, as compared to just the olfactory epithelium and lamina propria of the nasal cavity in intranasally exposed mice, at d1 pi, indicating neural invasion [51]. Furthermore, the viral antigen was readily present in the majority of mice within the nasal cavity, olfactory nerve, teeth, olfactory bulb, frontal cortex, and midbrain by d3 pi, unlike intranasally infected mice where presence of the antigen was sparse at d3 pi [51].

Subcutaneous routes of infection result in delayed CNS invasion, as compared to aerosol and intranasal routes of exposure to EEEV. Mice infected subcutaneously with EEEV present with a biphasic clinical illness characterized by an acute self-limiting replication phase in the periphery followed by lethal invasion of the CNS [38]. In the periphery, EEEV was found to replicate within fibroblasts and osteoblasts, whereas upon CNS invasion, the viral antigen was found to be randomly and widely dispersed throughout the brain, especially in neurons in the cerebral cortex, hippocampus, and midbrain, with the exception of the olfactory neuroepithelium, suggesting a vascular route of viral dissemination [38]. Importantly, the very-low-density lipoprotein receptor (VLDLR), which serves as a receptor for EEEV [13,14,15], was identified to play a key role in pathogenesis, as VLDLR KO mice had significantly reduced mortality as compared to WT mice upon subcutaneous exposure (Table 3), likely due to reduced infection, confirming that VLDLR is an important host factor for EEEV [58]. Lesions within the hippocampus were found to be more prominent in the dentate gyrus, a region of the brain which is particularly important for memory function. As such, damage to this region of the brain could impair the ability to form new memories, disrupt learning processes, and alter emotional regulation, all of which are implicated in human survivors of EEEV infection presenting with neurological sequelae. Additionally, within the hippocampus, the pyramidal neurons of the ventrolateral Cornu Ammonis 3 (CA3) field were implicated, and this region plays a critical role in forming and retrieving memories, spatial navigation, and emotional regulation [59]. Pyramidal neurons in this region are important for synaptic plasticity, and the virus targeting these neurons could point to why EEEV infection may lead to memory impairments or other cognitive deficits in survivors [60].

Subcutaneous models are also extremely useful in terms of studying naturally acquired infections and host immune responses. The main differences in subcutaneous models as compared to aerosol and intranasal models are the time it takes for the virus to reach the CNS, as it must first establish infection in the periphery, which also allows time for the host immune response to become activated and could explain why only a percentage (16%) of mice had detectable virus in the brain between days 3 and 6 pi [50] via the subcutaneous route of exposure. Moreover, subcutaneous models develop lymphopenia, similar to what is observed in humans and NHPs, as well as viremia, suggesting that subcutaneous models accurately recapitulate disease, with the caveat being that less mice overall develop CNS disease; however, this could be challenge dose-dependent. Another contributing factor to EEEV virulence is the presence of four binding sites within the viral 3′UTR for the hematopoietic cell-specific microRNA, miR-142-3p, which upon binding results in suppressed viral translation (Table 3) [61]. Combinatorial mutations of the EEEV miR-142-3p binding sites resulted in an attenuated phenotype in mice following subcutaneous challenge, confirming the importance of miR-142-3p for virulence in vivo [61]. Additionally, subcutaneous models allow for a longer therapeutic window before overt CNS disease; however, the distinction must be made as to whether the therapeutic and/or the host immune response is responsible for clearing the infection, which can be difficult to distinguish. Aerosol and intranasal exposures result in the virus being detected as early as 6 h post-infection and 1-day post-infection, respectively, thus limiting the therapeutic window for intervention. However, aerosol and intranasal routes of exposure are almost guaranteed to induce severe CNS disease, making them the gold standard for testing therapeutic and vaccine candidates.

Studies in both animal models and human autopsy specimens have shown extensive inflammation in the brain, such as perivascular cuffing, microglial activation, cytokine and chemokine expression, edema, and gliosis, likely exacerbating neuronal damage [4,44,45,47,49,53,62]. When comparing intranasal and aerosol routes of exposure, no significant differences were observed among any of the 25 cytokines and chemokines tested at any timepoint, suggesting these routes of infection induce similar immune responses [50]. Several differences in cytokine and chemokine expression were noted between subcutaneous and intranasal or aerosol routes of infection, including an increase in serum IFN-γ, macrophage inflammatory protein-1 beta (MIP-1β) (a chemoattractant for macrophages and natural killer (NK) cells), regulated upon activation, normal T-cell expressed and secreted (RANTES) (a chemokine for T-cells), and monokine-induced gamma interferon (MIG) (a T-cell attractant and activator) at day 1 pi in subcutaneous infected mice, followed by a return to baseline at day 2 pi, upon which the same cytokines/chemokines become increased in aerosol or intranasal infection. In macaques, there was a one-hundred- to one-thousand-fold increase in expression of traumatic brain injury genes like *LIF* and *MMP-9* and inflammatory cytokines and chemokines in the brain tissues [49]. MMP-9 is an enzyme that plays a role in the breakdown and remodeling of the extracellular matrix and is often associated with neuroinflammation, and LIF is a cytokine involved in the regulation of immune responses and neurogenesis [63,64,65,66]. This further suggests that the host inflammatory response to infection likely contributes to the neuropathogenesis and disease outcomes.

## 4. Venezuelan Equine Encephalitis Virus (VEEV)

### 4.1. Geography, Epidemiology, Clinical Disease

VEEV is mainly found in South and Central America as well as in southern states of the US, such as Florida and Texas [67]. VEEV is the least lethal among the encephalitic alphaviruses, with a less than 1% mortality rate in humans and 20–80% mortality in equines. Generally, encephalitis manifestation with VEEV is less common than with EEEV at about 14%, but 4–14% of survivors suffer from neurological sequelae following disease. Associated neurological sequelae for VEEV can include convulsions, somnolence, confusion, photophobia, coma, intellectual disability, and emotional instability/behavioral changes (Table 1). VEEV was first isolated from humans during an outbreak in southern Colombia in 1950 and from the brain of an infected horse in Venezuela in 1938 [68,69]. However, the virus had been affecting horses earlier, with the first well documented outbreak in equids occurring in the central river valleys of Colombia in 1935. This outbreak spread to Venezuela the following year and reached Trinidad by 1943 [70]. By the end of 1969, more VEEV outbreaks were reported in El Salvador and Guatemala, and eventually throughout most of Central America and Mexico, resulting in around 52,000 human cases, 50,000 horse deaths, and 93 human fatalities. The outbreak continued to spread, reaching the Golf Coast and southern Texas in 1971 [68]. In Texas, more than 1500 horses died, 110 humans were infected, and hundreds of VEEV isolates were obtained from field-caught mosquitoes [68]. The largest human outbreak occurred in Venezuela and Colombia in 1995, with 100,000 human cases, 3000 cases of neurological complications, and 300 deaths. This outbreak also resulted in 50,000 infected horses, with 4000 equine deaths reported [68,70]. Recent outbreaks were reported in Peru in 2005 and 2006 [68]. Assessing the full burden of VEEV in developing countries remains difficult, as it is clinically indistinguishable from other arboviral diseases such as dengue so cases may be under and/or misdiagnosed. The lack of widespread access to confirmatory diagnostic tests means that the extent of endemic disease is largely unknown [69]. It is estimated that between 0.1 and 7% of dengue cases in Latin America are actually cases caused by VEEV infection [71].

### 4.2. Animal Models, CNS Disease, and Neuropathology

Much of what is known about VEEV neuropathology has been determined through animal models. The most well characterized animal models are rodent and NHP models (Table 4). Most studies have utilized Cynomolgus macaques to study VEEV, as the model closely resembles human disease. Unfortunately, limited detailed pathological and histological studies have been performed for VEEV in NHPs [5], with most recent studies utilizing fever and viremia analysis to assess the responsiveness to vaccines and therapeutics [72,73]. Subcutaneous infection of Cynomolgus macaques results in a very mild disease, with fever and viremia observed, but no neurological disease is observed [4]. Cynomolgus macaques infected via aerosol with VEEV also have fever, viremia, and lymphopenia, but neurological symptoms are present including tremors, twitching, ataxia, photophobia, hyperactive/reactive, and nystagmus [72,74]. At day 6 post-infection, multiple indicators of inflammation were noted including increased levels of T-cells, elevated levels of MCP-1 and CXCL10 chemokines in the cerebral spinal fluid (CSF), traumatic brain injury markers *MMP-9* and *LIF* in the thalamus, and activated microglia [74]. Interestingly, at 4 weeks post-infection, although the macaques had physiologically recovered from VEEV infection, they displayed signs of neurological sequelae. MCP-1 and CXCL10 remained elevated in the CSF, viral RNA persisted in the CNS, and vasculitis and leukocyte infiltration were observed throughout the CNS. These studies indicate that Cynomolgus macaques are an important model to study neurological disease and sequelae following VEEV infection.

VEEV infection of mice results in a biphasic disease, with virus replicating in the periphery including lymphoid tissues, followed by CNS invasion. Infection of immunocompetent mice (e.g., CD-1, C57BL/6, BALB/c) with BSL-3 strains of VEEV results in severe encephalitic disease and mortality in the majority of mice [7,75]. Infection of C3H/HeN mice with VEEV TC-83, a live attenuated vaccine strain of VEEV, also causes severe disease and mortality and has great utility for antiviral testing at a reduced biosafety level [76]. In contrast, VEEV TC-83 infection of C57BL/6 mice results in low mortality rates (0–20% depending on viral dose), but mice still suffer from encephalitic disease [20,77,78,79]. Studies using this model have begun to elucidate neurological sequelae in surviving mice. Mice displayed deficits in prepulse inhibition (the inability to filter out extraneous sensory stimuli) [79]. Histopathological analysis of brain sections indicated that symptomatic mice had thalamus damage (glial nodules and calcification). Increased glial fibrillary acidic protein (GFAP) expression, an indicator of astrocyte activation, was observed in the thalamus and the hippocampus [77,79] and increased ionized calcium binding adaptor molecule 1 (Iba1) expression, an indicator of microglia activation, was seen in the hippocampus [77,79]. Interestingly, VEEV TC-83 infection of Tg2576 mice, a mouse model of Aβ amyloidosis used to study Alzheimer’s disease, resulted in more severe deficits such as glial cell activation, increased inflammatory cytokines in the brain, and increased latency to avoid and escape shock [77].

**Table 4:** VEEV animal models.

Animal Model		Viral Strain	Infection Route	% Mortality	Clinical Observations	Neuropathological Features	Refs.
Species	Strain						
NHP	Cynomolgus macaques	TrD, INH-9813	AE *	0%	Viremia, fever dehydration, lymphopenia, lethargy, dehydration, Ataxia, photophobia, nystagmus, tremors, hyperactivity, twitching	Vasculitis, leukocyte infiltration, lyphoplasmatic meningoencephalitis, gliosis	[72,74,80]
NHP	Cynomolgus macaques	INH-9813	SC **	0%	Fever, viremia	None	[4]
Mice	C3H/HeN,	TC83	IN ***	80% [76], 60% [81]	Weight loss, ruffled fur, viremia, hunched, lethargic	Perivascular cuffing, meningitis, encephalitis, gliosis	[76,81]
Mice	C57BL/6	TC83	IN	10–20%	Weight loss, ruffled fur, viremia	Glia cell activation, prepulse inhibition	[20,77,78,79]
Mice	C57BL/6, BALB/c, NIH Swiss	TrD, ZPC-738	IN	100%	Weight loss, viremia, ruffled fur, hunched posture, ataxia, altered gate, paralysis	Mononuclear cell infilaration, meningitis, encephalitis, microglia activation, neuron cell death	[82,83,84]
Mice	CD-1	V3000, INH9813	AE	100% with ≥10–100 PFU	Weight loss, ruffled fur, hunched, ataxia, serizures,	Not described	[52,85]
Mice	C57BL/6, CD-1, BALB/c	TrD, V3000, ZPC-738, INH9813	SC	100%	Weight loss, viremia, ruffled fur, hunched, lethargic, paralysis	Neuronal death, spongiosis, and gliosis	[52,54,86,87]

* AE: aerosol, ** SC: subcutaneous, *** IN: intranasal.

VEEV neuroinvasion in mice can occur through multiple routes. Following peripheral infection of mice, VEEV was found initially in the brain’s circumventricular organs (CVOs) that lack a BBB [21], suggesting that VEEV enters the brain via a hematogenous route bypassing the BBB. However, another study in mice showed that during peripheral infection VEEV enters the brain through caveolin-mediated transcytosis of brain endothelial cells across an intact BBB [88]. Mice deficient in caveolin-1 (Cav-1) had reduced viral titers in the brain, but not the periphery, indicating that Cav-1 plays a role in VEEV neuroinvasion [88] (Table 5). Intranasal infection of VEEV results in direct access of the virus to the brain via viral replication within olfactory sensory neurons in the olfactory neuroepithelium [5,83,89,90]. In either peripheral or intranasal infection, viral invasion into the CNS occurs prior to disruption of the BBB [20,88]. VEEV infection results in disruption of the BBB in a biphasic manner [91]. Loss of tight junction formation was observed at 6 days post-infection, whereas viral replication was detected as early as 1 day post-infection in the olfactory bulb. Interestingly, BBB disruption is at least partially mediated by toll-like receptor 4 (TLR4), as TLR4 knockout (KO) mice have decreased BBB permeability following VEEV TC-83 infection [92]. TLR4 KO mice did not display differences in viral titers or immune cells in the brain but had reduced gene expression of *MMP-9*, *MMP-2*, *ICAM-1*, C-C motif chemokine ligand 2 (*CCL2*), and *IFN-γ*. Another host factor that may be implicated in enhancing neuronal apoptosis post-infection with VEEV is early growth response 1 (EGR1). EGR1 is a transcription factor widely expressed in the brain and is upregulated post-infection with VEEV as determined via RNAseq [93].

Once VEEV enters the CNS, significantly pathology is observed throughout the brain. BALB/c mice infected subcutaneously with VEEV TrD had pathological lesions including spongiosis, gliosis, astrocytosis, and neuronal death in the meninges, olfactory bulb, isocortex, hippocampus, midbrain/thalamus, cerebellum, and pons, with the most severe pathology observed at day 6 post-infection [54]. Viral RNA was detected most prominently in the olfactory bulb and midbrain/thalamus but was also present in other regions, with neuropathology noted but to a lesser extent. Elevated levels of GFAP and Iba1 were noted, indicating glia cell activation and damage. In intranasally infected C57BL/6 mice, VEEV TC-83 is found in the olfactory bulb, cortex, cerebellum, brain stem, and spinal cord [20]. Infection progresses from the olfactory bulb to the back of the brain in a time-dependent manner, with peak VEEV replication detected in the olfactory bulb at 1–2 days post-infection, cortex at 2–6 days post-infection, cerebellum at 4–6 days post-infection, and brain stem at 4–6 days post-infection [20]. Similarly, intranasal infection of VEEV TC-83 in C3H/HeN mice showed infection of the main olfactory bulb, piriform cortex, striatum, motor cortex, thalamus, hippocampus, and cerebellum in a time-dependent manner [94].

Intranasal infection of C57BL/6J mice with VEEV ZPC-738 results in the rapid infection of olfactory sensory neurons [83]. LDLRAD3, a recently identified receptor of VEEV [16] (Table 5), is present in olfactory neurons [94], specifically in high levels within olfactory sensory neurons [83], suggesting that LDLRAD3 contributes to enhanced tropism of the olfactory sensory neurons. LDLRAD3 is essential for VEEV pathogenesis, as LDLRAD3 KO mice survive subcutaneous, intranasal, and intracranial challenges [86]. Peripheral challenge of LDLRAD3 KO mice with VEEV displayed decreased disease and viral dissemination [86]. LDLRAD3 is also important for infection of neurons, as intracranial inoculation of VEEV in LDLRAD3 KO mice showed limited infection in the brain and a significant reduction in mortality [86]. These results were recapitulated in vitro, as mixed cultures of primary mouse neurons and glial cells from cortexes of LDLRAD3 KO mice had reduced capability to support VEEV replication as compared to cultures from wildtype mice [86]. VEEV was still capable of replicating and causing limited disease in LDLRAD3 KO mice, indicating that there are additional receptors yet to be identified for VEEV.

VEEV predominantly infects neurons in vivo [5,20,76], but other cell types serve as minor infected cell populations or are impacted by the infection. Neuronal apoptosis is a feature observed across different VEEV mouse models, including the loss of olfactory neurons in VEEV TC-83-infected C3H/HeN mice [94]. Astrocyte activation has been noted both morphologically as well as by an increase in GFAP staining. VEEV replicates to high titers in microglia and astrocytes in vitro, but only a few studies have found evidence of VEEV-infected microglia and astrocytes recapitulated in mice [5,20,76]. However, microglia are activated following VEEV infection [94]. Microglia were found aggregated and wrapped around infected neurons [94], which is suggestive of them being involved in removal of dying neurons through efferocytosis. There is also some evidence that oligodendrocytes are infected by VEEV in mice [86,95]. Additional cells found within the CNS, including brain microvascular endothelial cells (BMECs), pericytes, ependymal cells, and choroid plexus, did not have detectable VEEV replication [5,20]. Viral replication in BMECs and pericytes was shown to be inhibited by type 1 IFN signaling [20], providing one mechanism by which replication is suppressed in these cells within the CNS.

Interferon α/β (IFNα/β), a critical antiviral component of the innate immune response, is important for controlling VEEV replication, but VEEV has multiple mechanisms to inhibit IFN induction, including capsid-mediated shutdown of host transcription [96]. IFNα/β receptor (IFNAR) KO mice infected subcutaneously with VEEV V3032, an attenuated strain of VEEV, had increased mortality compared to parental 129Sv/Ev mice [97]. In mice intranasally infected with VEEV ZPC-378, interferon stimulated gene (ISG) expression was delayed up to 48 h post-infection in the olfactory neuroepithelium and olfactory bulb, enabling VEEV infection to be established in these mice [83]. Multiple studies have tested if interferon treatment is a viable therapeutic option with mixed results. Treatment of mice with polyethylene glycol (PEG)-conjugated IFNα 24 h prior to subcutaneous infection with VEEV TrD protects mice from death [98]. In agreement with these results, prophylactic treatment of C3H/HeN mice intranasally infected with VEEV TC-83 resulted in a significant increase in survival [76]. However, treatment of IFNα either concomitantly, 1 h post-infection, or 3 h post-infection was unable to increase animal survival rates in mice intranasally infected with VEEV ZPC-738 [83]. It is possible that treatment with IFNα is still a viable option, as mice had induced ISG expression in olfactory sensory neurons, delayed onset of encephalitic symptoms and viral invasion in the CNS, and increased animal survival time [83].

Adaptive immune cells have been shown to contribute to disease severity in lethal mouse models. Severe combined immunodeficiency (SCID) mice that lack both T- and B-cells have reduced disease severity and an increase in the average survival time following subcutaneous infection with virulent VEEV V3000 [99]. CD4+ T-cells were shown to be essential for protection against lethal VEEV infection [100]. The impact of T- and B-cells has also been studied in non-lethal models of VEEV infection. Infection of mice lacking B-cells (uMT mice) with attenuated VEEV V3533 resulted in reduced viral replication in the brain and persistent infection as compared to Rag KO mice, which lack functional T- and B-cells, which displayed severe disease and succumbed to infection with VEEV V3533 [101]. Wildtype and αβ-T-cell receptor (TCR) KO mice infected intranasally with VEEV TC-83 had similar viral loads and disease symptoms during the first 2 weeks of VEEV infection [102]. However, αβ-TCR KO mice had persistent infection until 92 days post-infection, along with elevated brain cytokines (RANTES and MCP-1) and inflammatory infiltrates, indicating that T-cells are required to clear VEEV infection from the brain and for resolution of disease [102].

**Table 5:** Host factors involved in VEEV neuroinvasion and/or pathogenesis.

Host Protein	Function	Impact on VEEV Pathogenesis	Ref.
iNOS	Enzyme that produces nitric oxide (NO), which can be a neurotoxin	Increased survival time in iNOS KO mice	[103]
TNFα	Proinflammatory cytokine	Increased survival time in TNFα KO mice	[103]
Cav-1	Main component of caveolae, which are specialized structures in the plasma membrane that facilitate transport	Decreased neuroinvasion in Cav-1 KO mice	[88]
TLR4	Pattern recognition receptor	Decreased BBB permeability in TLR4 KO mice	[92]
LDLRAD3	Entry receptor for VEEV	Decreased viral replication and 100% survival in LDLRAD3-KO mice	[86]
ICAM-1	Adhesion molecule involved in leukocyte migration at the BBB	Decreased neuroinflammation (perivascular cuffing and neuronal necrosis) in ICAM-1 KO mice	[104]
IFNα/β	Antiviral cytokine	Increased mortality in IFNAR KO mice	[97]

Significant inflammation is observed in the brain after VEEV infection as evidenced by perivascular cuffing, meningitis, encephalitis, gliosis, and astrocytosis. Neuronal apoptosis is observed both in regions of the brain where the VEEV antigen is detected as well as regions lacking VEEV antigen but where astrogliosis is present [103]. This suggests that neuroinflammation contributes to neuronal death. Multiple studies have found alterations in inflammatory gene expression and cytokine protein levels in the brains of VEEV-infected mice [54,76,87,104]. Genes involved in the virus response, inflammatory response (e.g., *CXCL10*, *CXCL11*, *CCL5*), antigen presentation, and apoptosis were differentially expressed in VEEV-infected mice [87]. Some notable altered genes included interferon regulatory factor 7 (*Ifr7*), interferon alpha-inducible protein 27 (*Ifi27*), 2′-5′ oligoadenylate synthetase 1B (*Oas1b*), macrophage migration inhibitory factor (*Mif*), Clusterin, and major histocompatibility complex (MHC) class II. Multiple cytokines are upregulated in C3H/HeN mice intranasally infected with VEEV TC-83, including IL-1α, IL-1β, IL-6, IL-12, MCP-1, IFN-γ, TNF-α, MIP-1α, and RANTES [76]. Peak cytokine expression was observed at days 6 and 7 post-infection, which corresponds to the peak of viral replication and neuropathology. The proinflammatory mediators nitric oxide synthase (*iNOS*) and *TNFα* were also upregulated in the brains of VEEV-infected mice [103]. Both iNOS and TNFα at least partially contribute to disease, as iNOS and TNF receptor knockout mice had increased survival times as compared to wildtype mice (Table 5). In addition, multiple extracellular matrix and adhesion molecule genes, including *ICAM-1*, *VCAM-1*, *CD44*, cadherins, integrins, *MMPs* and tissue inhibitor of metalloproteinases 1 (*Timp1*), were upregulated in VEEV-infected mice [104]. ICAM1 KO mice had no differences in animal survival but showed decreased neuroinflammation and downregulation of proinflammatory cytokines/ligands including *CCL24*, *CXCL11*, and *CXCL13* (Table 5) [104]. While there were numerous inflammatory mediators altered in VEEV-infected mice, a correlation analysis found that TNF-α, CCL-2, CCL-5, and leukocyte infiltrates correlated with pathology in VEEV TrD-infected mice [54].

More recent studies have provided brain region-specific bulk RNAseq, spatial RNAseq, and single-cell RNAseq (scRNAseq) analyses of brain from VEEV-infected C3H/HeN mice [94]. RNAseq studies were performed on the olfactory bulb, piriform cortex, striatum, motor cortex, sensory cortex, hippocampus, thalamus, and cerebellum from VEEV-infected mice at days 1, 3, 5, 6, and 7 post-infection [94]. The most activated canonical pathways included the pathogen-induced cytokine storm signaling pathway, the macrophage classical activation signaling pathway, CREB signaling in neurons, dendritic cells and natural killer cells crosstalk, and neuroinflammation signaling. In addition, pyroptosis signaling and necroptosis signaling, both cell death pathways, were activated in VEEV-infected mice. Pathway activation increased over time and was generally consistent across brain regions. In a complementary study, spatial RNAseq and scRNAseq studies were performed using brain cerebral hemispheres collected at days 2, 4, and 6 post-infection [105]. The scRNAseq studies, performed on mononuclear immune cells, found an increase in microglia populations starting at day 2 post-infection that progressively increased over time. Infiltration of myeloid and lymphocyte (e.g., NK cells and T-cells) populations was observed at days 4 and 6 post-infection. Antiviral and inflammatory gene expression was observed in all cell types and increased as the infection progressed [105]. Spatial RNAseq demonstrated that infiltrating myeloid cells were found in specific areas of the brain. For example, myeloid cells that express lymphocyte antigen 6 family member C2 (*Ly6c2*) and placenta-specific 8 gene (*Plac8*) markers were found in the hippocampus and periphery of the cortex in VEEV-infected brains [105]. In contrast, infiltrating NK and CD8+ T-cells were localized throughout the brain parenchyma. NK and CD8+ T-cells were found to express high levels of genes encoding cytotoxic proteins, such as perforin, granzyme B, and *FasL*, at both day 4 and 6 post-infection, correlating with increased disease severity at these timepoints.

## 5. Western Equine Encephalitis Virus (WEEV)

### 5.1. Basic Background (Geography, Epidemiology, Clinical Disease)

WEEV is found primarily in the western regions of Canada and the US and the southern cone of South America [67,106]. WEEV is maintained in nature in an enzootic transmission cycle propagated by *Culex* and *Aedes* mosquitoes and birds and lagomorphs, which occasionally results in epizootic spillover into horses and humans [106]. Originally isolated from the brain of an infected horse during an outbreak in 1930 in California [107], WEEV has a case fatality rate of approximately 3–15% in humans and 3–50% in equines [67]. For WEEV, neurological sequelae occur in 15–30% of survivors and sequelae are similar to those described for EEEV and VEEV [1,67]. In infants one year old or less, 90% of cases result in severe CNS disease [5]. However, naturally acquired infections through the enzootic cycle typically result in 3–4% mortality, primarily affecting infants and elderly populations [108].

WEEV outbreaks were first described in the early to mid-20th century, with the largest WEEV outbreaks reported during the 1930s-1940s, where tens of thousands of equine and over 3000 human cases were reported [106]. Outbreaks of WEEV have significantly declined in frequency and scale, with less than 700 human cases documented between 1964 and 2009 and, as a result, a decrease in the mammalian virulence of contemporary WEEV isolates [17]. More recently, in November 2023, there was an outbreak of WEEV in Argentina and Uruguay, where a human patient in Argentina presented with sudden onset of fever and neurological features including headache, myalgia, dizziness, and disorientation. Upon receiving intensive medical care and mechanical ventilation for 12 days, the patient recovered [106,109,110]. This outbreak ultimately grew to 217 human cases, 12 of which were fatal, and 2548 equine cases [106,109,110]. Samples from confirmed horse WEEV clinical cases (*n* = 6), originating from outbreaks in 2023–2024 in Argentina, were analyzed for histological lesions within the brain. The primary regions affected were the brainstem (3/6), cerebellum (3/6), thalamus (5/6), cerebral cortex (6/6), and spinal cord (1/6) [110]. Fatal infections were determined to be driven primarily by infiltrating neutrophils and meningoencephalitis [110].

As with EEEV and VEEV, WEEV pathogenesis in humans and animals is largely dependent on age and route of infection [5]. Following infection, there is typically an incubation period ranging from 5 to 10 days with mild flu-like symptoms being observed in the majority of cases [7]. However, severe neurologic disease symptoms may manifest such as confusion, coma, weakness, drowsiness, and irritability, and as high as 15% of neurological cases result in fatality [7]. Moreover, approximately 15–30% of encephalitic case survivors suffer from significant neurological sequelae. Documented mild sequelae include loss of taste, changes in speech, loss of fine motor skills, abnormal gait, and hearing loss [111]. Case studies of severe WEEV-induced neurological sequelae in humans describe seizures, decreased motor skills, intellectual disability, learning disabilities, speech difficulties, psychological impairment, altered gait, taste distortion, and loss of facial movements in both adults and children, with a higher incidence of sequelae observed in children [111,112]. Neuropathological lesions documented in human cases of WEEV include perivascular cuffing of lymphocytes and neutrophils, multifocal necrosis, and gliosis. Both gray and white matter regions of the brain are affected including the basal nuclei, thalamus, brainstem, and spinal cord [5].

Encephalitic human cases of WEEV are characterized by the presence of vasculitis and focal hemorrhages in the basal ganglia and the nucleus of the thalamus [67]. In elderly patients, small hemorrhages are sometimes observed in the gray and white matter which may be mistaken for resolved infarcts [113]. Additionally, presence of perivascular cuffing in lymphocytes and neutrophils, multifocal necrosis, and gliosis has also been observed in clinical brain samples. Lesions in the spinal cord have also been observed [5].

### 5.2. Animal Models, CNS Disease, and Neuropathology

A limited number of NHP and mouse models of WEEV infection and disease have been studied (Table 6). As early as 1939, reports describing intranasal challenge of WEEV in rhesus macaques induced severe and lethal encephalitis [114]. Cynomolgus macaques were shown by Reed et al. to be a suitable model for aerosol exposure of WEEV and VEEV, with WEEV-exposed animals more likely to manifest neurological signs of encephalitis, including the presence of severe tremors [114]. Neuropathological observations with this model showed significant encephalitis characterized by perivascular cuffing and infiltrating neutrophils with antigen staining of microglia and neurons [114]. Another report described similar pathological findings in the CNS tissues of encephalitis or meningoencephalitis with the presence of perivascular cuffing in gray/white matter and meninges, gliosis, and viral antigen detected via IHC on day 8 post-infection in moribund animals [115]. Subcutaneous exposure of NHPs to WEEV resulted in no detectable CNS disease clinically or pathologically [4], suggesting that the aerosol route of exposure is more likely to induce CNS disease in NHP models of infection.

WEEV pathogenesis has been studied in numerous mouse strains (Table 6) including inbred and outbred strains of different ages and genders as well as various routes of exposure and challenge doses [108]. WEEV has been reported to infect inbred mice such as BALB/c and C57BL/6 as well as outbred strains of mice such as CD-1, among others. Blakely et al. confirmed that age, gender, and genetic background (strain) all have significant effects on disease susceptibility, independent of virus tropism or replication within the CNS, suggesting that experimental variables can be adjusted to recapitulate disease observed in NHPs and humans, which is imperative for vaccine and therapeutic development [116]. Interestingly, C57BL/6 mice between 10 and 15 weeks of age showed a significant drop in both clinical disease severity and mortality as compared to younger mice when subcutaneously infected with WEEV CBA-87. Moreover, the consistent disease observed in WEEV C57BL/6 mice, regardless of route, is ideal for future studies to include transgenic and gene knockout mice to aid in understanding the molecular drivers of pathogenesis and neurological sequelae.

Aerosol WEEV challenge studies in mice show that WEEV induces acute clinical disease that progresses to severe CNS disease in a dose-dependent manner when using highly lethal strains, such as Fleming and McMillan [108]. However, aerosol exposure with the less virulent strain Imperial 181 resulted in little overt clinical disease and complete survival in mice. Mice exposed to WEEV via aerosol have higher viral titers in the organs of the CNS and periphery, including spleen, lung, and salivary gland, as compared to mice challenged subcutaneously [117], suggesting that aerosol exposure is more neurovirulent in WEEV-infected mice. Moreover, mice subcutaneously infected with WEEV have higher viral titers in the knee, popliteal lymph node, and skeletal muscle, suggesting different tissue tropism based on the route of infection. In terms of clinical disease in aerosol models of WEEV, rapid onset of weight loss, lethargy, and labored breathing as well as neurological symptoms of disease including hyperreactivity, circling, seizures, and tremors, among others, are observed [108,118]. Following lethal aerosol challenge, pathological analysis revealed encephalitic focal lesions in the piriform cortex, presence of perivascular cuffing, mild focal gliosis, and pyknotic and karyorrhectic debris in some surrounding neurons at day 3 post-infection (pi). Meningoencephalitis with necrosis and degenerative changes in neurons and leukocytes in the brain was also observed on days 4 and 5 pi [108]. Another group described loss of gray matter and neuroparenchyma, necrosis, and presence of viral antigen in the cortex, hippocampus, thalamus, and hypothalamus as well as neurons of the ganglion layer, plexiform layers, and internal nuclear layers within the retina [108].

**Table 6:** WEEV animal models.

Animal Model		Viral Strain	Infection Route	% Mortality	Clinical Observations	Neuropathological Features	Refs.
Species	Strain						
NHP	Cynomolgus macaques	CBA-87, Fleming	AE *	9–100%	Fever, antisocial behavior, lethargy, reduced appetite, tremors	Encephalitis, occipital meningitis, focal hemorrhage in the frontal lobe and brainstem, perivascular cuffing and infiltrating neutrophils. Antigen in neurons and microglia of cortex.	[80,114,115]
NHP	Cynomolgus macaques	Fleming	SC **	0%	Minor weight loss	No lesions or viral antigen in the brain.	[4]
Mice	BALB/c	Fleming, McMillian (McM), Imperial 181 (IMP)	AE	0–100%	Weight loss, ruffled fur, hunched posture, lethargy, closed eyes, labored breathing, mobility issues. Hyperreactivity, circling seizures, tremors, spinning, fixed gaze, obsessive grooming, and/or twitching	Encephalitis, focal leptomeningitis in the cerebrum, perivascular cuffing, infiltrating neutrophils, gliosis, neuronal death. Antigen in neurons and neuronal processes of substantia nigra, CVOs, glial cells, and astrocytes.	[108,118]
Mice	CD-1, BALB/c, C57BL/6 and astrocyte specific NFκB KO mice	McM, McM-fLuc, CBA-87, Mn548, B11, Mn520	IN ***	100%	Ruffled fur, hunched posture, lethargy, labored breathing. Depression, motor deficits, ataxia, unresponsive to visual stimuli during late stage of infection, seizures	Necrosis and secondary demyelination in olfactory bulb, hypothalamus, midbrain, hippocampus; gliosis, infiltrating neutrophils, perivascular cuffing. Antigen in neurons of hippocampus and nigrostriatal pathway. Loss of neurons in hippocampus.	[119,120,121]
Mice	CD-1, C57BL/6 BALB/c, A/J, DBA/2, BALB/cBy	McM, IMP, CBA-87	SC	20–100% (McM, CBA-87), 0% (IMP)	Seizures, viremia	Meningoencephalitis in the hypothalamus, pineal gland, and the area postrema; perivascular cuffing and infiltrating neutrophils; necrosis in hippocampus, gliosis in frontal and cerebral cortex.	[21,116,117,122]

* AE: aerosol, ** SC: subcutaneous, *** IN: intranasal.

In intranasally infected mice, WEEV enters the brain through olfactory sensory neurons (OSNs) to directly induce CNS invasion [21]. Bioluminescent ex vivo imaging studies in mice intranasally infected with WEEV showed initial infection within the olfactory bulb and nasal turbinates which progressed along the lateral olfactory tract. The regions of the brain depicting high bioluminescence signal, which correlates with viral load, included the basal nuclei, thalamus, and hypothalamus. Intranasally infected mice have prominent viral antigens observed in the olfactory tract, whereas in subcutaneously infected mice, WEEV antigen is more observed within the caudoputamen region of the brain, an area that plays a critical role in motor, perceptual, and cognitive skills, as well as spatial working memory. Interestingly, CD-1 mice intranasally infected with WEEV had no detectable virus outside of the CNS [116], suggesting that viral replication in peripheral tissues is not required to induce fatal disease.

Differences in WEEV neuroinvasion have been noted between peripherally infected mice and intranasally infected mice. Subcutaneous mouse models of WEEV tend to be less neuroinvasive, in that a lesser proportion of mice develop CNS disease. Moreover, subcutaneous exposure results in viral CNS invasion through several routes and regions of the brain, including the bloodstream. Studies performed by Salimi et al. showed that peripheral infection of mice with WEEV resulted in infectious virus being detected at timepoints prior to disruption of the BBB [88]. Using confocal microscopy, WEEV antigen was detected in the brains’ circumventricular organs (CVOs), suggesting that WEEV enters the CNS by hematogenous seeding of the CVOs followed by centripetal spread along the neuronal axis [21]. Viral titers of CNS and periphery organs of mice subcutaneously infected with WEEV showed that the virus reached the CNS on day 2 pi, with infectious virus detected in the brainstem, spinal cord, and cerebrospinal fluid before reaching the cerebrum and cerebellum on day 3 pi, suggesting that CNS invasion occurs approximately 48 h prior to onset of clinical symptoms [21]. Interestingly, peak infection within the CNS occurs significantly before mice succumb to infection, suggesting that uncontrolled inflammation and immune response of the host are also contributing factors to death as a clinical outcome. Histological and histochemical features of CNS tissues showed a tropism for neurons, in line with what is observed in intranasal mouse models of WEEV [21]. Another study described meningoencephalitis in the hypothalamus, pineal gland, and the area postrema in mice subcutaneously infected with WEEV. Vascular congestion was observed in the meninges and corresponding parenchyma, with apoptosis evident by 72 to 96 h after neuroinvasion. These findings suggest that the virus invades the CNS following subcutaneous exposure, albeit slightly delayed compared to direct routes of exposure, and provides further evidence that subcutaneous exposure in mice mimics subcutaneous exposure in humans.

WEEV has been shown to be impacted by several host proteins, including Protocadherin-10 (PCDH10), VLDLR, Cav-1, and nuclear factor-kappa B (NF-κβ) (Table 7). Both PCDH10 and VLDLR can serve as receptors for WEEV [17,18]. VLDLR KO mice show decreased mortality following WEEV challenge and reduced viral RNA in the brain [58]. Similarly, decoys against PCDH10 or VLDLR protect from WEEV lethality [17,18]. Mutations in receptor PCDH10, which is widely expressed in the brain, have been linked to autism-spectrum disorders, obsessive–compulsive disorder, and depression [123], so this could be an interesting avenue to explore in the realm of prophylactic development to prevent neurological sequelae in survivors of encephalitic alphavirus infection. Another host factor implicated in WEEV (and VEEV) neuropathogenesis is Cav-1 [88]. WEEV and VEEV exploit Cav-1 mediated transcytosis to pass through an intact BBB, which may be regulated by IFN. Cav-1 KO mice had decreased viral titers in the brain as compared to WT mice during early timepoints following peripheral infection, suggesting that Cav-1 plays an important role in WEEV neuroinvasion [88]. However, lack of functional Cav-1 does not affect viral replication efficiency within the CNS, as Cav-1 KO mice intracranially inoculated with either virus had similar titers in the CNS as compared to WT mice [88].

Bantel et al. examined whether neuroinflammatory activation of glia is an initiating event in selective loss of dopaminergic neurons and α-synuclein protein aggregation following intranasal infection with WEEV [119]. Using astrocyte-specific NFκB KO mice, they saw a drastic reduction in dopaminergic neurons and α-synuclein aggregation post-infection with WEEV, suggesting that innate immune inflammatory signaling in astrocytes regulates both neuroinflammation and protein aggregation prior to neuronal injury [119] (Table 7). To prevent fatal encephalitis and allow for animal survival following infection with WEEV, immunotherapy was administered to WT mice (CD-1 and C57BL/6) and surviving mice showed significant changes in locomotion and gait activities, even at 8 weeks post-infection, suggesting sustained neuroinflammation as a consequence of viral infection is a contributing factor to the sequelae observed. Histopathological analysis of infected brains showed that WEEV antigen was heavily concentrated in neuronal cell populations in the entorhinal cortex and hippocampus, as well as dopaminergic neurons in the nigrostriatal pathway [119]. Importantly, microglia and astrocytes were not observed to contain any viral antigen, unlike previous findings describing moderate WEEV antigen detected in both astrocytes and oligodendrocytes [21], but in a subcutaneous mouse model. In another C57BL/6 model, IHC identified the WEEV capsid protein in neurons within the hippocampus. Infected cells were found throughout multiple brain regions, including the cerebellum [21].

**Table 7:** Host factors involved in WEEV neuroinvasion and/or pathogenesis.

Host Protein	Function	Impact on VEEV Pathogenesis	Ref.
Cav-1	Main component of caveolae, which are specialized structures in the plasma membrane that facilitate transport	Decreased neuroinvasion in Cav-1 KO mice	[88]
PCDH10	Cell adhesion molecule and entry receptor for WEEV	Treatment with PCDH10 decoy (PCDH10EC1–Fc) protects mice from WEEV lethal challenge	[17]
VLDLR	Entry receptor for WEEV	Increased survival in VLDLR KO mice	[58]
NFκB	Transcription factor that regulates inflammation and apoptotic signaling	Decreased α-synuclein aggregation, neuronal loss, and gliosis in astrocyte-specific NFκB KO mice	[119]

Treatment of animals with IFN-α protects from lethal challenge of WEEV [124], regardless of the timing of prophylaxis [125]. These findings are in line with mouse models of VEEV treated with IFNα conjugated to PEG, as described earlier [98]. Additionally, these findings suggest that immunomodulating therapy combined with broad spectrum antivirals, such as ribavirin in the case of Hepatitis C [126], may enhance clinical outcome. A limited number of host factors have been identified for WEEV, two for EEEV, and several for VEEV. The resurgence in interest in encephalitic alphaviruses [2] will hopefully yield more detailed host factor assessments that can be validated in animal models.

## 6. Discussion and Conclusions

Encephalitis in humans is clinically characterized by abrupt onset of fever, intense headache, irritability, restlessness, drowsiness, anorexia, nausea, vomiting, diarrhea, cyanosis, convulsions, and coma [50]. Animal models that accurately recapitulate human disease are of utmost importance when attempting to understand the molecular mechanisms underlying disease pathogenesis. NHPs are the most similar model to humans; however, although clinically relevant, NHP studies require extensive resources, equipment, training, and facilities. As such, mouse models are heavily utilized due to them being widely accessible. While mouse models cannot completely recapitulate human disease, mouse models do manifest lethargy, fever, and neurological symptoms, such as tremors, making them a suitable model for studying encephalitic alphaviruses. Moreover, using mouse models is advantageous in infectious disease research due to the ease of acquisition, low demand on resources, ease of handling for technical manipulations, and ease of acquiring sufficient number of samples to demonstrate statistical significance. Differences were observed in terms of viral dissemination and lesion severity between intranasally and aerosol-infected mice, with aerosol exposure resulting in more caudal regions of the brain being implicated, whereas intranasal routes of exposure are more, but not entirely, limited to the nasal cavity, olfactory epithelium and bulb, and hippocampus. Thus, the route of exposure needs to be considered when designing animal studies and choosing biomarkers of disease.

Neurons are a major target cell of encephalitic alphaviruses, especially within the substantia nigra, which plays a central role in Parkinson’s disease (PD) and aligns with descriptions of neurological sequelae symptoms reported to be similar to PD symptoms. Current insight suggests that persistent neuroinflammation and hyperactive signaling in glial cells resulting in loss of dopaminergic neurons within the substantia nigra are the major underlying mechanisms of PD neuropathology [119]. Interestingly, acupuncture has been shown to serve a neuroprotective, anti-inflammatory, and anti-apoptotic role in patients with PD [127]; however, more studies are warranted. It would be interesting to assess if natural remedies such acupuncture could prevent loss of dopaminergic neurons following infection with WEEV. Loss of dopaminergic neurons is driven by increased expression of TNFα. Previous studies have shown that TNFα is increased post-infection with encephalitic alphaviruses [88,93,128] and drives neuronal cell death. Recent studies using a subcutaneous mouse model of VEEV found TNFα, CCL-2, CCL-5, and leukocyte infiltration as biomarkers for severe neuropathology. Therapeutically targeting these biomarkers to dampen neuronal insults may yield a better clinical outcome; however, this has yet to be studied in vivo. Encephalitic alphaviruses have evolved to utilize multiple dynamic host factors, in addition to viral proteins, some of which may have redundant roles, to ensure viral replication. While significant progress has been made, more efforts are required to better understand additional underlying molecular markers and altered cell signaling pathways driving severe neuropathology and clinical outcomes.

As research progresses in the field of viral encephalitis, the utilization of animal models will become increasingly important for testing developing therapeutics and vaccines for efficacy. While there are several ethical and financial drawbacks that inherently come with using NHPs in research, NHP models offer invaluable insight when studying viral encephalitis. Aerosol or intranasal exposure elicits severe CNS disease in NHPs, similar to what is observed in human clinical cases of encephalitic alphaviruses. As such, under the Animal Rule, validation of therapeutic and vaccine efficacy will likely require NHP studies. As higher-order species are extremely valuable, future NHP work should include behavioral assessments pre- and post-infection to further aid in the understanding of long-term neurological consequences of infection and how this mimics sequelae observed in human cases. However, this may require using less pathogenic viral strains or lower challenge doses and/or immunotherapy treatment in order for the animals to survive acute infection.

Mice are widely used in all aspects of biomedical research, especially in infectious disease, as they provide insight into pathogenesis that cannot be captured in computer-generated models. Mice can be transgenically manipulated to express or knock-down certain genes of interest, which has certainly been shown to be valuable in delineating viral and host factors that enhance pathogenesis. Continued efforts to unravel viral and host factors underlying encephalitic alphaviruses are required, especially for EEEV and WEEV. Moreover, as these markers are uncovered, it would be interesting to examine if therapeutic targeting of these markers could potentially alleviate neuronal insults encountered during acute infection to prevent or reduce subsequent neurological sequelae. An established mouse model would ideally include a fully characterized spatiotemporal analysis of neuroinvasion and neurodegeneration within the brain throughout the course of infection. As evidenced in the literature, there are a variety of exposure routes from which CNS disease can develop. Careful consideration should be given to viral strain, viral dose, and challenge route utilized in order to develop an optimized animal model that accurately and reliably recapitulates neurological damage observed in humans.

Behavioral assays are well established in mice and will be useful in determining the presence and severity of neurological sequelae. For example, the elevated plus maze (EPM) is based on aversion to open spaces and is commonly used to assess anxiety in mice [129], while the Y-maze can be used to assess spatial working memory [130], which is regulated by the hippocampus, a region of the brain heavily impacted by encephalitic alphaviruses [129,130]. Another behavioral test which measures neuromuscular function is the rotarod test, which allows for long-term disability monitoring in surviving mice [131]. This test evaluates coordination, balance, and fatigue as mice attempt to keep balance on a turning rod, with neurologically impaired mice falling off the rod sooner than WT mice. Neuromuscular function following encephalitic alphavirus exposure can also be assessed using a modified SHIRPA approach [79]. Other behavioral testing paradigms that can be employed include the open field test, nest building test, resident intruder test, Barnes maze test, and repetitive grooming tests [132]. Many of these tests are used for studying PD, Alzheimer’s disease, Amyotrophic Lateral Sclerosis (ALS), Attention-Deficit/Hyperactivity Disorder (ADHD), and autism. Additionally, many neuropathological features described for these diseases are also observed in cases of viral encephalitis. It would be interesting to examine the compounding effects of preexisting conditions on viral encephalitis and subsequent neuropathology and neurological deficits. Moreover, there are some approved therapeutic options available for these conditions which could potentially be promising in limiting neuronal insults post-infection with encephalitic alphaviruses.

In conclusion, there are a variety of applicable NHP and mouse models available for studying encephalitic alphavirus neuropathogenesis. These animals develop CNS disease similar to humans and are undoubtedly the unsung heroes of biomedical research. Continued efforts of characterizing these models should include detailed neuropathological analysis. Recently developed technologies, such as scRNAseq and omics approaches, will aid tremendously in helping to unravel the tangled webs weaved by encephalitic alphaviruses.

## Figures and Tables

**Table 2:** EEEV animal models.

Animal Model		Viral Strain	Infection Route	% Mortality	Clinical Observations	Neuropathological Features	Refs.
Species	Strain						
NHP	Cynomolgus macaque, common marmoset	V105-00210	AE *	60–100%	Weight loss, fever, ataxia, nystagmus, lethargy, twitching, tremors, and seizures (lethal)	Meningoencephalitis and vasculitis, neuronal degeneration and necrosis, perivascular cuffs, cellular debris, gliosis, satellitosis, edema, hemorrhage, infiltrating neutrophils, and lymphocytic infiltrates within the hippocampus, amygdala, corpus striatum, thalamus, mesencephalon, and medulla oblongata.	[47,48,49]
NHP	Common marmoset	FL939-39	IN **	100%	Weight loss, anorexia, lethargy, hunched posture, somnolence	Encephalitis, mononuclear cell leptomeningitis, perivascular cuffing in cerebral cortex and hippocampus. Microglial activation and neuronal necrosis, viral antigen in cerebral cortex.	[42]
NHP	Cynomolgus macaque	FL939-39	SC ***	75%	Dehydration, ataxia, intermittent tremors, lethargy, difficulty standing on both feet, abnormal vocalizations, hyperthermia, excessive salivation, recumbency, facial edema, anorexia	Neuronal necrosis and gliosis in the cerebrum, inflammation in the meninges, and perivascular cuffing in the cerebrum and cerebellum. Acute inflammation in the cerebrum, cerebellum, and brain stem neuropil; chronic inflammation in the meninges, choroid plexus, and cerebral neuropil; gliosis in the brain stem; hemorrhage in the cerebrum and brainstem neuropil	[4]
Mice	BALB/c, CD-1	FL939-39, EEEV NA V105	AE	80–100%	Weight loss, ataxia, ruffled fur, hunched posture, lethargy, dehydration, head tilt, circling, fixed gaze, paralysis, lateral recumbency, and seizures (rare)	Meningoencephalitis; Neuronal vacuolation, spongiosis, necrosis. Viral antigen in nasal cavity, olfactory bulbs, frontal cortex, hippocampus, thalamus, midbrain, cerebellum, brain stem, spinal cord, and pituitary gland.	[50,51,52,53]
Mice	BALB/c	FL939-39	IN	Not described [50,51]	Viremia, weight loss, ataxia, ruffled fur, hunched posture, lethargy, dehydration, head tilt, circling, fixed gaze, paralysis, lateral recumbency, and seizures (rare)	Neuronal cell death in the hippocampus. Viral antigen in olfactory epithelium, olfactory bulb, neurons in the cerebrum, frontal cortex, and midbrain.	[50,51]
Mice	BALB/c, C57BL/6	FL939-39	SC	100% [38], Not described [50,51]	Ruffled fur, hunched posture, lethargy, tremors, lateral recumbency	Neuronal apoptosis, necrosis, spongiosis, meningoencephalitis, vasculitis, thrombosis, and perivascular cuffing. Viral antigen in olfactory epithelium, cortex, hippocampus, caudate putamen, thalamus.	[38,50,51]

* AE: aerosol, ** IN: intranasal, *** SC: subcutaneous.

**Table 3:** Host factors involved in EEEV neuroinvasion and/or pathogenesis.

Host Factor	Function	Impact on EEEV Pathogenesis	Ref.
VLDLR	Entry receptor for EEEV	Increased survival in VLDLR KO mice	[58]
miR-142-3p	Hematopoietic cell-specific microRNA	Increased survival following infection with combinatorial mutations of EEEV miR-142-3p	[61]

## Data Availability

Not applicable.
